# Multiple ocular manifestations in a case of cat scratch disease without systemic signs

**DOI:** 10.3205/oc000172

**Published:** 2020-12-15

**Authors:** Kanako Annoura, Ichiya Sano, Shinji Makino, Hidetoshi Kawashima

**Affiliations:** 1 Department of Ophthalmology, Jichi Medical University, Shimotsuke-shi, Japan

**Keywords:** cat scratch disease, anterior uveitis, serous macular detachment

## Abstract

A 43-year-old woman presented with impaired vision and redness in her left eye of 2 weeks duration. She had a pet cat that scratched her forehead 3 weeks before she presented to us. She had no systemic signs such as lymphadenopathy, fever, or fatigue. The respective right and left corrected visual acuities were 20/16 and 20/2000. The anterior chamber of the left eye exhibited inflammation; a fundus examination of that eye revealed optic disc swelling and a serous macular detachment with hard stellate exudates. Based on the recent cat scratch and the ocular findings, cat scratch disease (CSD) was suspected. The results of serologic testing showed elevated titres of IgM and IgG antibodies to *Bartonella henselae*. Administration of doxycycline and a steroid was initiated. This report describes the occurrence of multiple ocular manifestations of CSD in both the posterior and the anterior segment.

## Introduction

Cat scratch disease (CSD), a rare infection caused by *Bartonella henselae*, typically presents as subacute lymphadenopathy, fever, malaise, chills, and small skin lesions following a cat scratch or bite. Ocular involvement occurs in 5% to 10% of infected patients [1]. The clinical manifestations of ocular involvement include Parinaud oculoglandular syndrome (POGS), neuroretinitis, serous retinal detachment, retinal infiltrates, choroiditis, branch retinal vessel occlusion, endophthalmitis, and anterior uveitis. The presence of ocular symptoms without systemic CSD is uncommon, as are fundus manifestations in patients with POGS or anterior uveitis [2]. We report a rare case of CSD in which there were multiple ocular manifestations but no systemic signs.

## Case description

**Figure 1:**
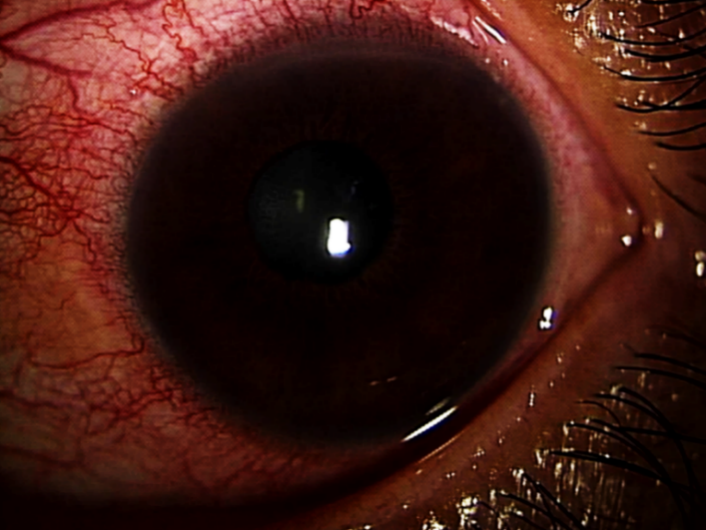
The chemosis with serous discharge

**Figure 2:**
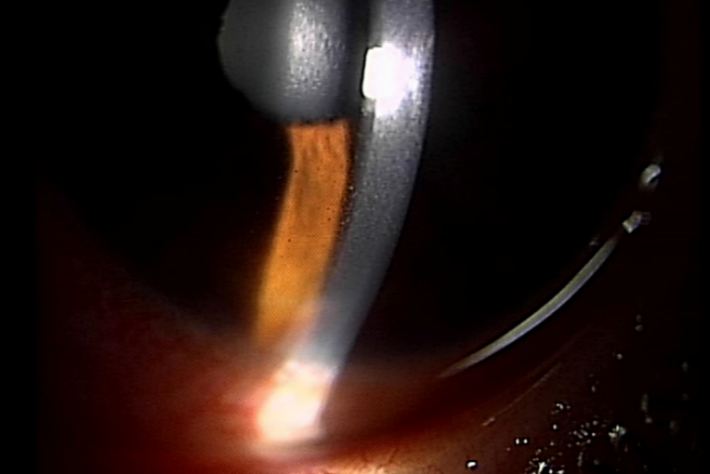
Keratic precipitates and cells in the anterior chamber

**Figure 3:**
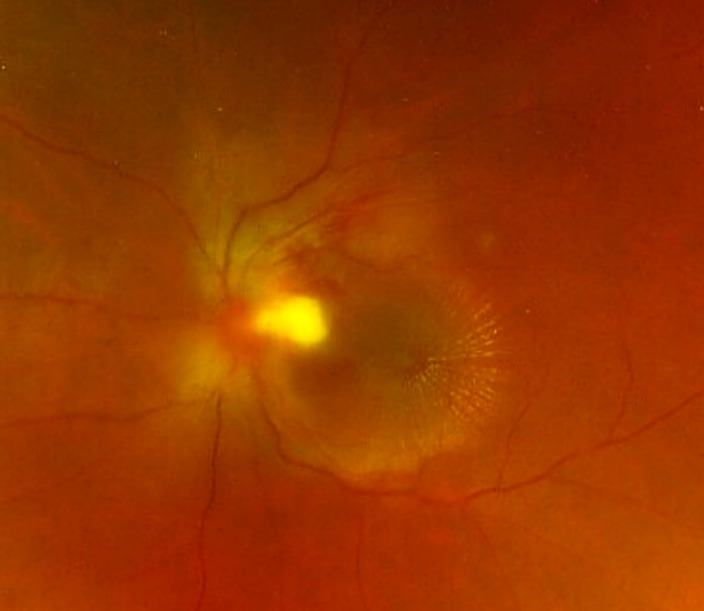
Optic disc swelling, a focal retinitis lesion at the temporal margin of the optic disc, macular exudates in a star pattern, and a flame-like retinal haemorrhage

**Figure 4:**
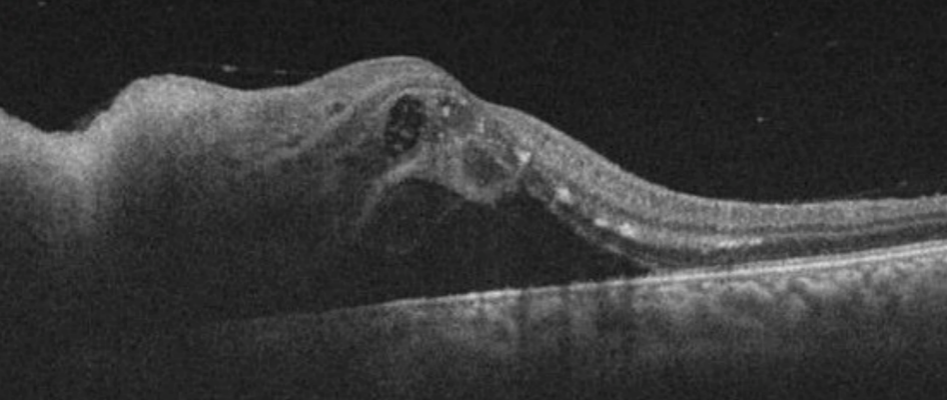
A serous macular detachment

**Figure 5:**
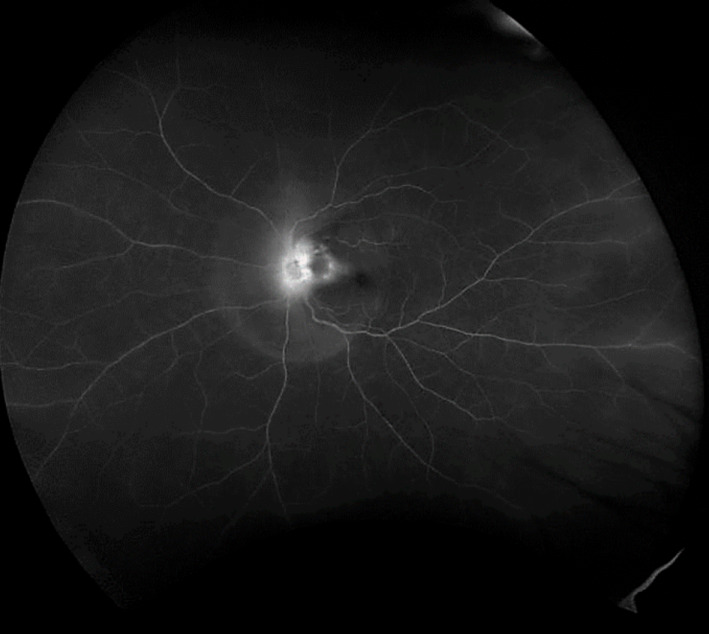
Leakage from the optic disc of the left eye (fluorescein angiography)

A 43-year-old woman presented with impaired vision in her left eye for the previous 2 weeks. She had a non-traumatic unilateral red eye, chemosis, and untreated hypertension. At the initial visit, the respective right and left corrected visual acuities were 20/16 and 20/2000, and the bilateral intraocular pressure was 20 mm Hg. The chemosis was accompanied by serous discharge, keratic precipitates, partial synechia of the iris and cells (2+) in the anterior chamber (Figure 1 (Fig. 1), Figure 2 (Fig. 2)), and vitreous of the left eye. A fundus examination of the left eye showed optic disc swelling, a focal retinitis lesion at the temporal margin of the optic disc, macular exudates in a star pattern, a flame-like retinal haemorrhage, and a serous macular detachment (Figure 3 (Fig. 3), Figure 4 (Fig. 4)). Fluorescein angiography showed leakage from the optic disc of the left eye (Figure 5 (Fig. 5)). No signs of inflammation were apparent in the right eye. The patient reported that her pet cat had scratched her forehead 3 weeks before she presented for examination. She did not have a fever, any detectable skin lesions, or lymphadenopathy. An infectious disease specialist performed a systemic examination and found no evidence of systemic CSD or any other infectious disease.

Since conjunctivitis and iridocyclitis were present in addition to the aforementioned fundus findings, extensive investigations for uveitis were conducted that included measurement of the total blood count and erythrocyte sedimentation rate (ESR) as well as tests for autoantibodies, syphilis, viruses, and toxoplasma antibody. Except for a slightly elevated ESR rate (26 mm/hour), the results were unremarkable. Angiotensin-converting enzyme, soluble interleukin-2 receptor, and chest x-ray parameters were all within the normal limits. A blood sample was sent to a pathology laboratory for serology testing for *B. henselae* antibody.

Since optic disc edema is associated with a macular star, it is important to exclude other causes such as malignant hypertension, sarcoidosis, toxoplasmosis, tuberculosis, and spirochetal disease [2]. The systemic blood pressure was 160/91 mm Hg, and all blood test results were within the normal limits, with the exception of the slightly elevated ESR. QuantiFERON TB (Qiagen, Germantown, MD, USA) was negative. Serology testing for *B. henselae* antibodies was the only positive test result (IgM titre 1:16, IgG titre 1:256). Serology tests for cytomegalovirus, toxoplasma, and human immunodeficiency virus were negative.

Given the clinical ocular findings and the reported cat scratch, CSD was suspected even without systemic illness. Oral doxycycline 200 mg/day and prednisolone 20 mg/day with tapering were prescribed. Topical corticosteroid and mydriatic treatments were administered for the anterior chamber inflammation.

**Figure 6:**
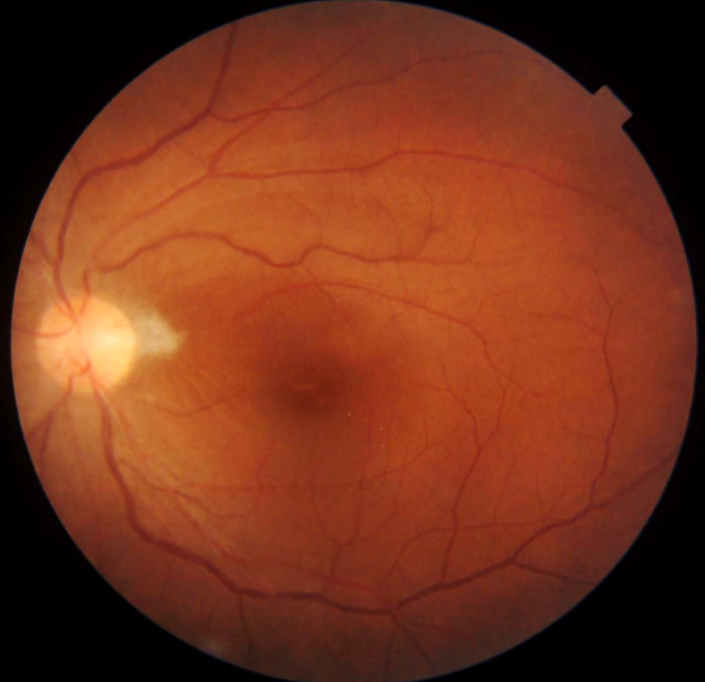
After the treatment, optic disc edema, focal retinal infiltrates, retinal haemorrhage, serous retinal detachment, and macular exudates decreased.

After the initiation of treatment, the optic disc edema, focal retinal infiltrates, retinal haemorrhage, serous retinal detachment, and macular exudates gradually decreased (Figure 6 (Fig. 6)). The cells in the anterior chamber and vitreous resolved over the treatment course. However, it is noteworthy that the focal retinitis persisted and doxycycline was prescribed for 3 months. Six months after the start of treatment, macular atrophy developed and the visual acuity remained 20/40.

## Discussion

Three aspects of the current case are particularly noteworthy. One pertains to various complications involved. POGS is reportedly the most common of the potential ocular manifestations of CSD, occurring in approximately 5% of patients with CSD [1]. Others include neuroretinitis (1–2%), and, less frequently, choroiditis, uveitis, retinal branch artery occlusion, retinal vein occlusion, and serous retinal detachment [3]. Even though each of these individual manifestations are rare in CSD, the current patient exhibited all of these ocular manifestations in the current case. POGS, anterior uveitis, neuroretinitis, a serous retinal detachment, and a flame-like retinal haemorrhage indicating retinal vein occlusion were all detected. POGS usually presents as unilateral conjunctivitis with or without conjunctival granulomata [4]. Although granulomatous lesions in the conjunctiva were not detected in the current patient, chemosis and conjunctival injection with serous discharge were seen. Simultaneous neuroretinitis is uncommon in patients with POGS [5], and anterior uveitis occurring in association with CSD is also rare [6]. In a previously reported case in a child, repetitive anterior uveitis was followed by neuroretinitis [7]. Thus, the simultaneous occurrence of anterior and posterior complications are noteworthy. Another notable feature of the current case was that the ocular involvement manifested without systemic disorders.

The current case is interesting in that neither lymphatic disease nor systemic symptoms developed. Classical CSD presents as a febrile illness associated with lymphadenopathy, skin lesions, and fever. Lymphadenopathy has been considered a diagnostic criterion of CSD [8], [9]. The presence of ocular symptoms without systemic CSD is unusual, although some cases with ocular symptoms but no systemic illness have been reported [10]. The current patient had no evidence of systemic disease. We suspected CSD based on her report of a relatively recent cat scratch and various ocular findings in conjunction with negative laboratory test results related to other possible diseases.

Unfortunately, the prognosis was not good despite the fact that the condition was most likely benign. Since CSD is regarded as benign, it is not uncommon for patients to be observed without treatment. Treatment remains controversial due to the self-limiting nature of the disease. Neuroretinitis usually follows a benign course and visual function recovers in just a few months [11]. However, notably, ocular CSD can be accompanied by complications resulting in permanent visual loss. A strong clinical suspicion of CSD as the cause of vision-threatening intraocular inflammation is necessary before initiation of prompt antibiotic treatment. Reports have suggested that early treatment can improve visual outcomes [11], [12]. Since the current patient exhibited severe ocular complications, we prescribed both antibiotic and corticosteroid treatments. The efficacy of systemic corticosteroids remains controversial, but in patients with severe inflammation, systemic corticosteroids should be beneficial. Despite the early intervention and the long treatment duration in the current patient, the visual acuity recovery was not as good as we anticipated.

## Conclusion

Ocular CSD is associated with various findings in the anterior and posterior segments, but simultaneous anterior and posterior complications are rare. We report a rare case of CSD in which there were simultaneous multiple ocular manifestations but no systemic signs. CSD should be considered in patients with ocular signs, even in the absence of systemic signs.

## Notes

### Competing interests

The authors declare that they have no competing interests.
